# Associations between blood‐based biomarkers of Alzheimer’s disease and rate of global and domain‐specific cognitive decline in an elderly population with 15 years of follow‐up

**DOI:** 10.1002/alz.087382

**Published:** 2025-01-03

**Authors:** Erika J Laukka, Ingrid Ekström, Giulia Grande, Martina Valletta, Chengxuan Qiu, Bengt Winblad, Claudia Fredolini, Laura Fratiglioni, Davide Liborio Vetrano

**Affiliations:** ^1^ Aging Research Center, Karolinska Institutet and Stockholm University, Stockholm Sweden; ^2^ Karolinska Institutet, Stockholm Sweden; ^3^ Theme Inflammation and Aging, Karolinska University Hospital, Stockholm Sweden; ^4^ Division of Neurogeriatrics, Karolinska Institutet, Stockholm Sweden; ^5^ SciLifeLab, Stockholm Sweden; ^6^ Aging Research Center and Center for Alzheimer Research, Karolinska Institutet‐Stockholm University, Stockholm Sweden

## Abstract

**Background:**

Alzheimer’s disease (AD)‐related biomarkers assessed in blood have proved to be useful for detecting dementia and cognitive impairment in the memory clinic. However, less is known about their predictive value in the long‐term and in a community‐based setting.

**Method:**

We examined six common serum‐derived biomarkers of AD (P‐tau181, T‐tau, Aβ42/Aβ40 ratio, P‐tau181/Aβ42 ratio, Nfl, and GFAP) from the baseline assessment of the population‐based Swedish National Study on Aging and Care in Kungsholmen (SNAC‐K; *n* = 2071, mean age at baseline = 72 years). At baseline, participants were free from dementia and neurodegenerative disorders and their cognitive level was within normal range (Mini‐Mental State Examination score > 23). Global cognition and performance in four specific domains (i.e., episodic and semantic memory, verbal fluency, and perceptual speed) were assessed regularly with a neuropsychological test battery across up to 15 years. Associations of AD‐related serum biomarkers with rate of cognitive decline across the follow‐up period were examined with linear mixed‐effects models.

**Result:**

Effects on rate of change in all examined cognitive domains were observed for all biomarkers, except T‐tau. The strongest associations were observed for P‐tau181 (global cognition: β = ‐0.024, 95% CI: ‐0.029 to ‐0.020) and for individuals with relatively high biomarker concentrations (3^rd^ or 4^th^ quartile). Interactions were explored for age, sex, and *APOE* ԑ4‐carrier‐status.

**Conclusion:**

To summarize, this study shows that higher concentrations of blood‐based biomarkers for neurodegeneration are associated with faster rates of cognitive decline in the general elderly population.

